# Conservation profile of endemic species of *Berberis* from Ecuador (Berberidaceae, Ranunculales)

**DOI:** 10.3897/BDJ.13.e157827

**Published:** 2025-07-07

**Authors:** Carmen Ulloa Ulloa, Enmily Sánchez-Lara, Nora H. Oleas

**Affiliations:** 1 Missouri Botanical Garden, St. Louis, United States of America Missouri Botanical Garden St. Louis United States of America; 2 Universidad Tecnológica Indoamérica, Quito, Ecuador Universidad Tecnológica Indoamérica Quito Ecuador

**Keywords:** Andes, Berberidaceae, extinction risks, IUCN assessment, páramos

## Abstract

**Background:**

The plant family Berberidaceae is represented in Ecuador by a single genus, *Berberis*. It comprises 15 species, seven endemic to the high Andean forests and páramos above 2,400 meters of altitude. These ecosystems, globally recognized for their exceptional biodiversity, are increasingly threatened by fragmentation and degradation, placing endemic species at serious risk of extinction. The conservation status of *Berberis* species in Ecuador was last assessed more than 20 years ago, underscoring the urgent need for a comprehensive and updated evaluation.

**New information:**

This study presents IUCN Red List assessments for all endemic species of the genus *Berberis* in Ecuador. Incorporating the latest taxonomic revision, we evaluate *Berberisengleriana* and *B.rigida* for the first time. Among the five species previously assessed, three have retained their original conservation status, while two are now classified under a higher threat category. Conservation measures are proposed to mitigate extinction risk and support the preservation of these species.

## Introduction

The páramos and cloud forests of the Andes rank among the most diverse regions on Earth, hosting a wealth of endemic species (Pérez-Escobar et al. 2022, Raven et al. 2020). The cloud forests of the Ecuadorian Andes are estimated to contain approximately 3028 endemic plant species, representing nearly 67.5% of the country's total endemic species (Jost 2011). The Ecuadorian páramos, meanwhile, are home to 659 endemic species, the result of evolutionary processes such as diversification, geographic isolation, and adaptive radiation (León-Yánez 2011).

This remarkable diversity is largely attributed to the geological formation of the Andes, which has created a unique assortment of physical, climatic, and habitat conditions along altitudinal gradients (Lozano et al. 2020). However, the reduction in size, fragmentation, and ongoing degradation of these ecosystems have left endemic species facing high rates of extinction and threat (Kipkoech et al. 2019). Climate change represents a significant threat to the conservation of endemic species. Many of these species are highly specialized, adapted to small ecosystems with specific microclimates, so any rapid alteration in weather patterns can have serious consequences for their survival. In the Ecuadorian Andes, vegetation is fragmented and separated by large areas of crops, creating physical barriers that hinder natural plant migration. These barriers limit species ability to adapt to new conditions, increasing their risk of extinction (Daehler 2005, León-Yánez 2011).

The Berberidaceae plant family consists of 13 to 19 genera and approximately 700 species, most of which belong to the genus *Berberis* L. (Hsieh et al. 2022). While Berberidaceae species are primarily distributed across the Northern Hemisphere, the only genus native in the Southern Hemisphere is *Berberis*, with its presence extending in South America along the Andes, from Venezuela to Chile and Argentina, and one species in the south of Brazil, and rare in tropical Africa. In Ecuador, the genus *Berberis* includes 15 species, seven of which are endemic (Ulloa Ulloa and Oleas 2024).

Species in this genus are shrubs or small trees that typically grow in shrubby *páramos* (a moorland type of high tropical Andean vegetation) and high-altitude Andean forests above 2,400 meters of altitude. All species in South America have simple, often spiny leaves and bear pale yellow to orange flowers, either solitary or in clusters. Their fruits are black-purple berries. While several species from this family are cultivated as ornamental plants in temperate regions of the Northern Hemisphere, none are grown as ornamentals in Ecuador, although they are often left as spiny live hedges along pastures (Ulloa Ulloa and Oleas 2024).

This study aims to assess the conservation status of all endemic *Berberis* species in Ecuador, a genus that inhabits the highly diverse yet increasingly threatened Andean ecosystem. By incorporating the latest taxonomic revisions and evaluating species previously unassessed, this research provides updated IUCN Red List assessments and highlights changes in threat levels. Additionally, it proposes conservation measures to mitigate extinction risks and support the long-term preservation of these endemic species.

### Distribution area

**Figure 1.**
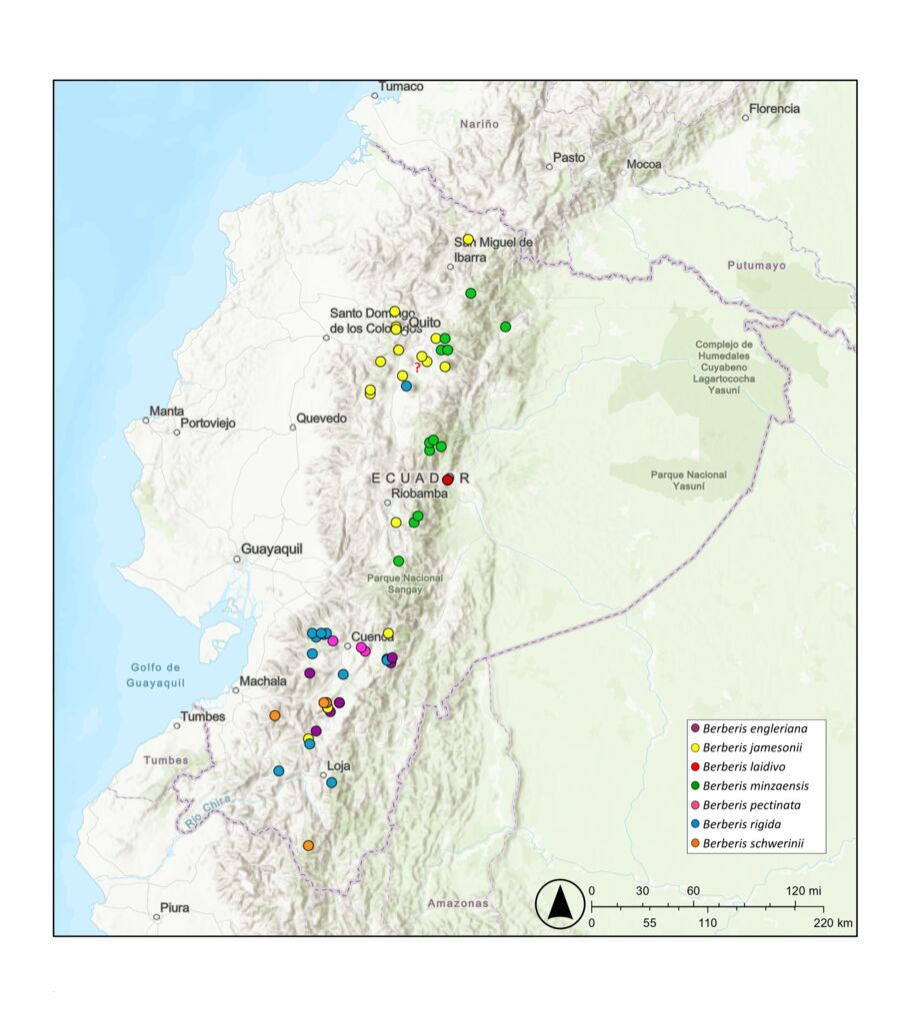
Distribution of *Berberis* species. Localities are based on herbarium specimens.

The genus *Berberis* in South America is distributed along the Andes from Venezuela to Chile and Argentina, and extending into southeastern Brazil (Ahrendt 1961). In Ecuador, 15 species have been reported, seven of which are endemic. These species are distributed throughout the Andean *páramos* and montane forests (Ulloa Ulloa and Oleas 2024). Among the endemic species in Ecuador, *B.jamesonnii* shows the widest distribution, followed by *B.minzaensis*. Most of the endemic *Berberis* species in Ecuador are restricted to the southern Andes. Fig. 1

## Materials and Methods

Information on *Berberis* was compiled for an exhaustive taxonomic revision for the Flora of Ecuador series from scientific literature, herbarium collections, and online records (Ulloa Ulloa and Oleas 2024). Specimens were studied by Ulloa Ulloa during visits to the following herbaria: A, AAU, BM, C, F, G, GB, GOET, GH, HA, HUH, HUTI, HUTPL, K, LD, LOJA, M, MA, MO, NY, P, QCA, QCNE, S, US, and W; selected loans were studied later with Oleas at MO.

The geographical distribution of the species was determined through a review of herbarium specimens and records available in globally accessible databases, such as the Missouri Botanical Garden TROPICOS® botanical database. To generate the species distribution map, all location records were georeferenced using Google Earth to ensure maximum spatial accuracy. The coordinates were then imported into ArcMap 10.8.2 and overlaid on the ArcGIS Desktop 10.8.2 basemap, using the appropriate projection settings for Ecuador to ensure spatial consistency and accurate representation. The conservation status of the species was evaluated following the IUCN Red List categories and criteria (IUCN 2012). Geospatial analysis was conducted using the Geospatial Conservation Assessment Tool (GeoCAT) (Bachman et al. 2011). The Area of Occupancy (AOO) was calculated with a cell width of 2 km, as recommended by IUCN (2012).

## Results

We provide an updated IUCN Red List assessment of seven *Berberis* species endemic to Ecuador. We evaluate *Berberisengleriana* and *B.rigida* for the first time. Among the five species previously assessed, three have retained their original conservation status, while two are now classified under a higher threat category. A summary of the assessments is available in Table 1.

## Species Conservation Profiles

### Berberis engleriana

#### Species information

Scientific name: Berberisengleriana

Species authority: C.K. Schneid.

Common names: Shuspilla

Kingdom: Plantae

Phylum: Streptophyta

Class: Equisetopsida

Order: Ranunculales

Family: Berberidaceae

Taxonomic notes: *Berberisengleriana* is easily recognized by the pale yellow flowers and spiny leaves with broad, nearly circular blades with olive-green upper surfaces.

**Figure 2.**
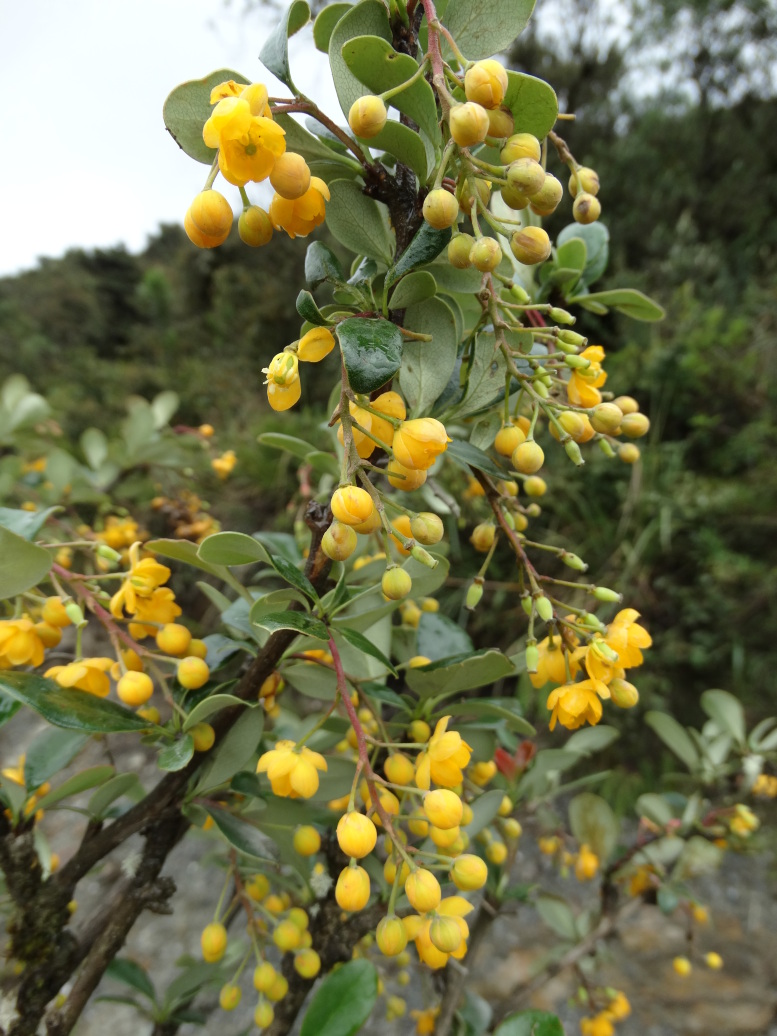
*Berberisengleriana* at Azuay, Ecuador. Ulloa, C 2520. Photo FloraoftheWorld courtesy Carmen Ulloa.

Figure(s) or Photo(s): Fig. 2

Region for assessment: Global

#### Editor & Reviewers

##### Editor

Editor: Ulloa Ulloa, C; Sánchez, E. & Oleas, N.H.

#### Geographic range

Biogeographic realm: Neotropical

Countries: Ecuador

Map of records (image): Fig. 1

Map of records (Google Earth): none

Basis of EOO and AOO: Observed

Basis (narrative): Unknown

Min Elevation/Depth (m): 3350

Max Elevation/Depth (m): 3650

Range description: The species is endemic to southern Ecuador. It is distributed in four provinces: Cañar, Azuay, Loja, and Morona Santiago.

#### Extent of occurrence

EOO (km2): 2327

Trend: Unknown

Causes ceased?: Unknown

Causes understood?: Unknown

Causes reversible?: Unknown

Extreme fluctuations?: Unknown

#### Area of occupancy

Trend: Unknown

Causes ceased?: Unknown

Causes understood?: Unknown

Causes reversible?: Unknown

Extreme fluctuations?: Unknown

AOO (km2): 32

#### Locations

Number of locations: 8

Justification for number of locations: The species has been documented in eight locations across the provinces of Cañar, Azuay, Loja, and Morona Santiago.

Trend: Unknown

Extreme fluctuations?: Unknown

#### Population

Trend: Unknown

Causes ceased?: Unknown

Causes understood?: Unknown

Causes reversible?: Unknown

Extreme fluctuations?: Unknown

#### Subpopulations

Number of subpopulations: 4

Trend: Unknown

Justification for trend: The subpopulations are geographically isolated across the provinces of Cañar, Azuay, Loja, and Morona Santiago. Limited genetic exchange between these subpopulations is likely, and they face potential threats from urban development and road construction, which could lead to their eventual disappearance.

Extreme fluctuations?: Unknown

Severe fragmentation?: Unknown

#### Habitat

System: Terrestrial

Habitat specialist: Yes

Habitat (narrative): It lives in the moorland and cloud forest of Ecuador.

Trend in extent, area or quality?: Decline (observed)

##### Habitat

Habitat importance: Major Importance

Habitats: 1. Forest1.9. Forest - Subtropical/Tropical Moist Montane

#### Ecology

Size: Unknown

Generation length (yr): 0

Dependency of single sp?: Unknown

Ecology and traits (narrative): Terrestrial

#### Threats

Justification for threats: Continuing decline of Habitat. Populated settlements and road construction, however, could be a potent threat, although the extent of this reduction cannot be estimated with certainty.

##### Threats

Threat type: Ongoing

Threats: 1. Residential & commercial development1.1. Residential & commercial development - Housing & urban areas1.2. Residential & commercial development - Commercial & industrial areas1.3. Residential & commercial development - Tourism & recreation areas4. Transportation & service corridors4.1. Transportation & service corridors - Roads & railroads6. Human intrusions & disturbance6.1. Human intrusions & disturbance - Recreational activities6.3. Human intrusions & disturbance - Work & other activities

#### Conservation

Justification for conservation actions:

Habitat protection, ongoing monitoring, community education and engagement, and scientific research.

**IUCN Red List assessment**

Due to its limited extent and occupancy (EOO of 2327 km², AOO of 32 km²) the small number of locations (8), the fragmentation of subpopulations, and current and future threats, the species can be classified as '**Endangered' (EN)** according to criterion **B1ab (iii) + B2ab (iii).**

It is recommended that habitat trends and potential threats continue to be monitored to determine whether the species should be re-evaluated in another category in the future.

##### Conservation actions

Conservation action type: Needed

Conservation actions: 1. Land/water protection2. Land/water management2.1. Land/water management - Site/area management3. Species management3.1. Species management - Species management5. Law & policy5.1. Law & policy - Legislation

#### Other

##### Use and trade

Use type: National

Use and trade: 18. Unknown

##### Use and trade

Use type: International

##### Use and trade

Use type: International

##### Ecosystem services

Ecosystem service type: Very important

##### Research needed

Research needed: 1. Research1.2. Research - Population size, distribution & trends1.5. Research - Threats3. Monitoring3.1. Monitoring - Population trends3.3. Monitoring - Trade trends3.4. Monitoring - Habitat trends

Justification for research needed: The species *Berberisengleriana* has been registered as a new endemic species for southern Ecuador, so its exact localities and population size have not been studied or documented.

### Berberis jamesonii

#### Species information

Scientific name: Berberisjamesonii

Species authority: Lindl.

Common names: Espuela

Kingdom: Plantae

Phylum: Streptophyta

Class: Equisetopsida

Order: Ranunculales

Family: Berberidaceae

Taxonomic notes: *Berberisjamesonii* is characterized by the minute reticulate venation on the upper leaf surfaces and dense inflorescence with numerous orange-yellow flowers.

**Figure 3.**
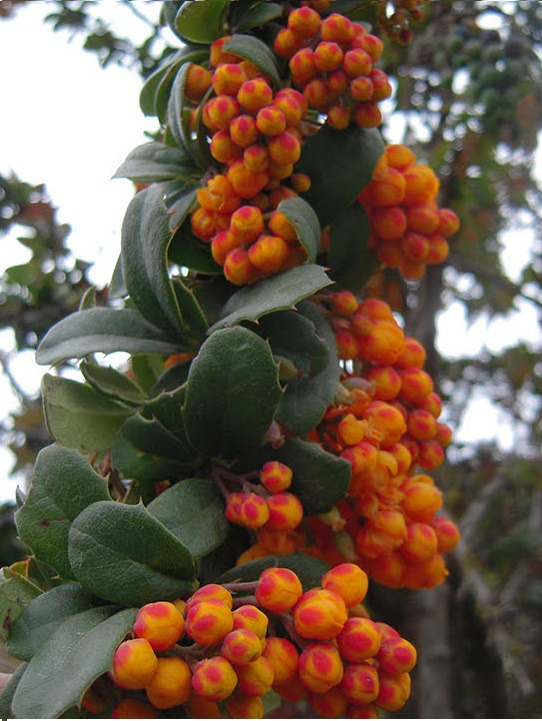
*Berberisjamesonii* at Pichincha, Ecuador. Ulloa, C 2430. Photo courtesy Carmen Ulloa.

Figure(s) or Photo(s): Fig. 3

Region for assessment: Global

#### Editor & Reviewers

##### Editor

Editor: Ulloa Ulloa, C; Sánchez, E. & Oleas, N.H.

#### Geographic range

Biogeographic realm: Neotropical

Countries: Ecuador

Map of records (image): Fig. 1

Map of records (Google Earth): none

Basis of EOO and AOO: Observed

Basis (narrative): Unknown

Min Elevation/Depth (m): 2500

Max Elevation/Depth (m): 3000

Range description: Endemic species of Ecuador. The herbarium collections come from the Quebrada del Río Ángel in Carchi; from the La Joya Imbabura road; Calacalí–Nono road, La Occidental road, Machachi road, Cerro Rumiñahui, Píntag–Antisana road, Hacienda Pinantura; Lloa of Pichincha road; Toacazo–Sigchos of Cotopaxi road; from Chimborazo; from Cañar; Paute road in Azuay; from Fierro Urcu in Loja; and from the slopes of the Antisana volcano from the province of Napo.

#### Extent of occurrence

EOO (km2): 39145

Trend: Unknown

Causes ceased?: Unknown

Causes understood?: Unknown

Causes reversible?: Unknown

Extreme fluctuations?: Unknown

#### Area of occupancy

Trend: Unknown

Causes ceased?: Unknown

Causes understood?: Unknown

Causes reversible?: Unknown

Extreme fluctuations?: Unknown

AOO (km2): 64

#### Locations

Number of locations: 13

Justification for number of locations: The species is documented in 13 locations distributed among the provinces of Carchi, Imbabura, Pichincha, Cotopaxi, Chimborazo, Azuay, Cañar, Loja and Napo.

Trend: Unknown

Extreme fluctuations?: Unknown

#### Population

Trend: Unknown

Causes ceased?: Unknown

Causes understood?: Unknown

Causes reversible?: Unknown

Extreme fluctuations?: Unknown

#### Subpopulations

Number of subpopulations: 15

Trend: Unknown

Justification for trend: New records have been included for the species in different geographical areas, so the number of subpopulations has increased since the last evaluation, where at least five widely dispersed populations were known.

Extreme fluctuations?: Unknown

Severe fragmentation?: Unknown

#### Habitat

System: Terrestrial

Habitat specialist: Yes

Habitat (narrative): It lives in the grassland and forest subtropical of Ecuador.

Trend in extent, area or quality?: Decline (observed)

Justification for trend: Continuing decline in area, extent and/or quality of habitat.

##### Habitat

Habitat importance: Major Importance

Habitats: 1. Forest4. Grassland4.7. Grassland - Subtropical/High Altitude

#### Ecology

Size: Unknown

Generation length (yr): 0

Dependency of single sp?: Unknown

Ecology and traits (narrative): Terrestrial

#### Threats

Justification for threats: Continuing decline of Habitat. Habitat destruction is the only known threat to the species (Ulloa Ulloa and Pitman 2003e).

##### Threats

Threat type: Ongoing

Threats: 1. Residential & commercial development2. Agriculture & aquaculture4. Transportation & service corridors6. Human intrusions & disturbance7. Natural system modifications

#### Conservation

Justification for conservation actions:

Its existence is confirmed within the Los Ilinizas Ecological Reserve and the Pululahua Geobotanical Reserve.

**IUCN Red List assessment**

The species range is very small (64 km²), a continuing decline in habitat area, extent, and/or quality is known, and habitat destruction is cited as the only known threat. This could become a significant problem in the future. Therefore, we could recognize the taxon as '**Near Threatened' (NT).**

##### Conservation actions

Conservation action type: Needed

Conservation actions: 2. Land/water management2.1. Land/water management - Site/area management5. Law & policy5.1. Law & policy - Legislation

##### Conservation actions

Conservation action type: Needed

Conservation actions: 3. Species management3.1. Species management - Species management

##### Conservation actions

Conservation action type: In Place

##### Conservation actions

Conservation action type: In Place

#### Other

##### Use and trade

Use type: National

Use and trade: 18. Unknown

##### Ecosystem services

Ecosystem service type: Very important

##### Research needed

Research needed: 1. Research1.2. Research - Population size, distribution & trends1.5. Research - Threats3. Monitoring3.1. Monitoring - Population trends3.3. Monitoring - Trade trends

Justification for research needed: In order to better understand population dynamics, it is crucial to gather more detailed information of population size, the number of mature individuals, and habitat and ecology that can be used to identify changes over time.

### Berberis laidivo

#### Species information

Scientific name: Berberislaidivo

Species authority: L.A. Camargo

Common names: Laidivo

Kingdom: Plantae

Phylum: Streptophyta

Class: Equisetopsida

Order: Ranunculales

Family: Berberidaceae

Taxonomic notes: *Berberislaidivo* is only known from two herbarium collections that match well in leaf characters: a sterile specimen collected in 1924 (*Tate 458*, deposited at the US herbarium) and a fertile one collected in 1943 (*Acosta Solis 6277*, deposited at the F herbarium); both collections have stiffly, coriaceous, sharply spiny-toothed leaves, which are shiny above and dull below. The collection at F, serving as type, has a coarse, erect inflorescence. This species has not been collected in the last 80 years.

Region for assessment: Global

#### Editor & Reviewers

##### Editor

Editor: Ulloa Ulloa, C; Sánchez, E. & Oleas, N.H.

#### Geographic range

Biogeographic realm: Neotropical

Countries: Ecuador

Map of records (image): Fig. 1

Map of records (Google Earth): none

Basis of EOO and AOO: None

Basis (narrative): Not available

Min Elevation/Depth (m): 3000

Max Elevation/Depth (m): 4000

Range description: Species of shrub endemic to Ecuador. The herbarium collections come from two locations, one in “Chaparro de Gualicón” (Bolívar province) and the other in “Sinche”, presumably in the province of Cotopaxi. The coordinates are unknown for these collections.

#### Extent of occurrence

EOO (km2): Not available

Trend: Unknown

Causes ceased?: Unknown

Causes understood?: Unknown

Causes reversible?: Unknown

Extreme fluctuations?: Unknown

#### Area of occupancy

Trend: Unknown

Causes ceased?: Unknown

Causes understood?: Unknown

Causes reversible?: Unknown

Extreme fluctuations?: Unknown

AOO (km2): 8

#### Locations

Number of locations: 2

Justification for number of locations: The species is documented in only two locations; one is in the Bolívar province and the other in Sinche, possibly Cotopaxi Province. There are no further reference data (Ulloa Ulloa and Pitman 2003d).

Trend: Unknown

Extreme fluctuations?: Unknown

#### Population

Trend: Unknown

Causes ceased?: Unknown

Causes understood?: Unknown

Causes reversible?: Unknown

Extreme fluctuations?: Unknown

#### Subpopulations

Number of subpopulations: 2

Trend: Unknown

Extreme fluctuations?: Unknown

Severe fragmentation?: Unknown

#### Habitat

System: Terrestrial

Habitat specialist: Yes

Habitat (narrative): Grows in the humid grasslands.

Trend in extent, area or quality?: Unknown

##### Habitat

Habitat importance: Major Importance

Habitats: 4. Grassland4.7. Grassland - Subtropical/High Altitude

#### Ecology

Size: Unknown

Generation length (yr): 0

Dependency of single sp?: Unknown

Ecology and traits (narrative): Terrestrial

#### Threats

Justification for threats: The exact threat events for this species are unknown.

##### Threats

Threat type: Ongoing

Threats: 12. Other options - Other threat

#### Conservation

Justification for conservation actions:

Habitat protection, ongoing monitoring, community education and engagement, and scientific research.

**IUCN Red List assessment**

We have no data on extent of occurrence (EOO), area of occupancy (AOO), number of mature individuals, or subpopulation status. Therefore, it remains in the '**Data Deficient' (DD)** category due to high uncertainty about any serious future threat that could cause a rapid decline of the species to EN or CR. The lack of detailed information on threats and population dynamics makes this assessment provisional and subject to revision as new data become available.

##### Conservation actions

Conservation action type: Needed

Conservation actions: 2. Land/water management2.1. Land/water management - Site/area management3. Species management3.1. Species management - Species management5. Law & policy5.1. Law & policy - Legislation

#### Other

##### Use and trade

Use type: National

Use and trade: 18. Unknown

##### Ecosystem services

Ecosystem service type: Very important

##### Research needed

Research needed: 1. Research1.2. Research - Population size, distribution & trends1.5. Research - Threats2. Conservation Planning2.1. Conservation Planning - Species Action/Recovery Plan3. Monitoring3.1. Monitoring - Population trends3.3. Monitoring - Trade trends

Justification for research needed: No specimens of this taxon are currently preserved in Ecuadorian herbaria. Therefore, it is recommended to conduct targeted investigations and additional collections of the species to better understand its distribution. Further research is needed to gather information on population dynamics, including fluctuations and size, the number of mature individuals and data on habitat and ecological condition. Also, monitoring changes over time will provide with valuable information for conservation strategies.

### Berberis minzaensis

#### Species information

Scientific name: Berberisminzaensis

Species authority: L.A. Camargo VU

Common names: Espino cruz

Kingdom: Plantae

Phylum: Streptophyta

Class: Equisetopsida

Order: Ranunculales

Family: Berberidaceae

Taxonomic notes: *Berberisminzaensis* is distinguished by its articulate petioles, conspicuously reticulate leaf venation and pubescent pedicels. It only occurs on the Eastern Cordillera.

Region for assessment: Global

#### Editor & Reviewers

##### Editor

Editor: Ulloa Ulloa, C; Sánchez, E. & Oleas, N.H.

#### Geographic range

Biogeographic realm: Neotropical

Countries: Ecuador

Map of records (image): Fig. 1

Map of records (Google Earth): none

Basis of EOO and AOO: Observed

Basis (narrative): Unknown

Min Elevation/Depth (m): 3500

Max Elevation/Depth (m): 4000

Range description: Endemic to Ecuador, it is registered in two protected areas of the country (Parque Nacional Cayambe-Coca and Parque Nacional Llanganates). The collections also come from Imbabura Province; others from Atillo in Chimborazo province; Laguna and Páramo de Papallacta in Napo province; Anuela River, Quijos, Páramo de Minza in Tungurahua province, and the Alao–Huamboya road in Morona Santiago province.

#### Extent of occurrence

EOO (km2): 7073

Trend: Unknown

Causes ceased?: Unknown

Causes understood?: Unknown

Causes reversible?: Unknown

Extreme fluctuations?: Unknown

#### Area of occupancy

Trend: Unknown

Causes ceased?: Unknown

Causes understood?: Unknown

Causes reversible?: Unknown

Extreme fluctuations?: Unknown

AOO (km2): 48

#### Locations

Number of locations: 10

Justification for number of locations: The species is documented in 10 locations distributed among the provinces of Imbabura, Pichincha, Tungurahua, Chimborazo, Napo, and Morona Santiago.

Trend: Unknown

Extreme fluctuations?: Unknown

#### Population

Trend: Unknown

Causes ceased?: Unknown

Causes understood?: Unknown

Causes reversible?: Unknown

Extreme fluctuations?: Unknown

#### Subpopulations

Number of subpopulations: 12

Trend: Increase

Justification for trend: Previously, at least four subpopulations were known in the northern Andes, but at the moment, other populations are recorded in the central provinces of the country.

Extreme fluctuations?: Unknown

Severe fragmentation?: Unknown

#### Habitat

System: Terrestrial

Habitat specialist: Yes

Habitat (narrative): In the humid and bushy moorland

Trend in extent, area or quality?: Decline (observed)

Justification for trend: Continuing habitat destruction. Continuing decline in area, extent and/or quality of habitat.

##### Habitat

Habitat importance: Major Importance

Habitats: 3. Shrubland3.7. Shrubland - Subtropical/Tropical High Altitude4. Grassland4.7. Grassland - Subtropical/High Altitude

#### Ecology

Size: Unknown

Generation length (yr): 0

Dependency of single sp?: Unknown

Ecology and traits (narrative): Terrestrial

#### Threats

Justification for threats: Habitat destruction is the only known threat to the species, caused by human development, industries and transportation (Ulloa Ulloa and Pitman 2003c).

##### Threats

Threat type: Ongoing

Threats: 1. Residential & commercial development1.1. Residential & commercial development - Housing & urban areas2. Agriculture & aquaculture3. Energy production & mining4. Transportation & service corridors6. Human intrusions & disturbance

#### Conservation

Justification for conservation actions:

A new record of this species was reported in 1983 (41688 MO) was obtained in the Laguna Encantada, located within the protected area of the Parque Nacional Llanganates (Ulloa Ulloa and Oleas 2024). This finding highlights the potential presence of rare and endemic species in protected areas, and the importance of these areas as key habitats for the conservation of biodiversity.

**IUCN Red List assessment**

Given that its extent of occurrence and occupancy area (EOO of 7073 km², AOO of 48 km²) are below the established threshold, there is continued habitat destruction, and the number of locations is 10, the species can be classified as '**Vulnerable' (VU)** under criterion **B1ab (iii) + B2ab (iii).** However, more data on population size and its declining trend would be needed to fully implement other criteria.

##### Conservation actions

Conservation action type: Needed

Conservation actions: 1. Land/water protection1.1. Land/water protection - Site/area protection2. Land/water management2.1. Land/water management - Site/area management3. Species management3.1. Species management - Species management5. Law & policy5.1. Law & policy - Legislation

#### Other

##### Use and trade

Use type: International

##### Ecosystem services

Ecosystem service type: Very important

##### Research needed

Research needed: 1. Research1.2. Research - Population size, distribution & trends1.5. Research - Threats2. Conservation Planning2.1. Conservation Planning - Species Action/Recovery Plan3. Monitoring3.1. Monitoring - Population trends3.3. Monitoring - Trade trends

Justification for research needed: It is necessary to carry out research projects on the population dynamics and ecology of the species. This would provide the greatest amount of information possible for a better evaluation supported by several criteria.

### Berberis pectinata

#### Species information

Scientific name: Berberispectinata

Species authority: Hieron.

Synonyms: *Berberiswarszewiczii*

Kingdom: Plantae

Phylum: Streptophyta

Class: Equisetopsida

Order: Ranunculales

Family: Berberidaceae

Taxonomic notes:

This species was previously treated as *Berberiswarszewiczii* Hieron., a name currently considered a synonym (Ulloa Ulloa and Oleas 2024).

*Berberispectinata* is characterized by the ciliate-spiny leaf margins and long-peduncled inflorescences.

**Figure 4.**
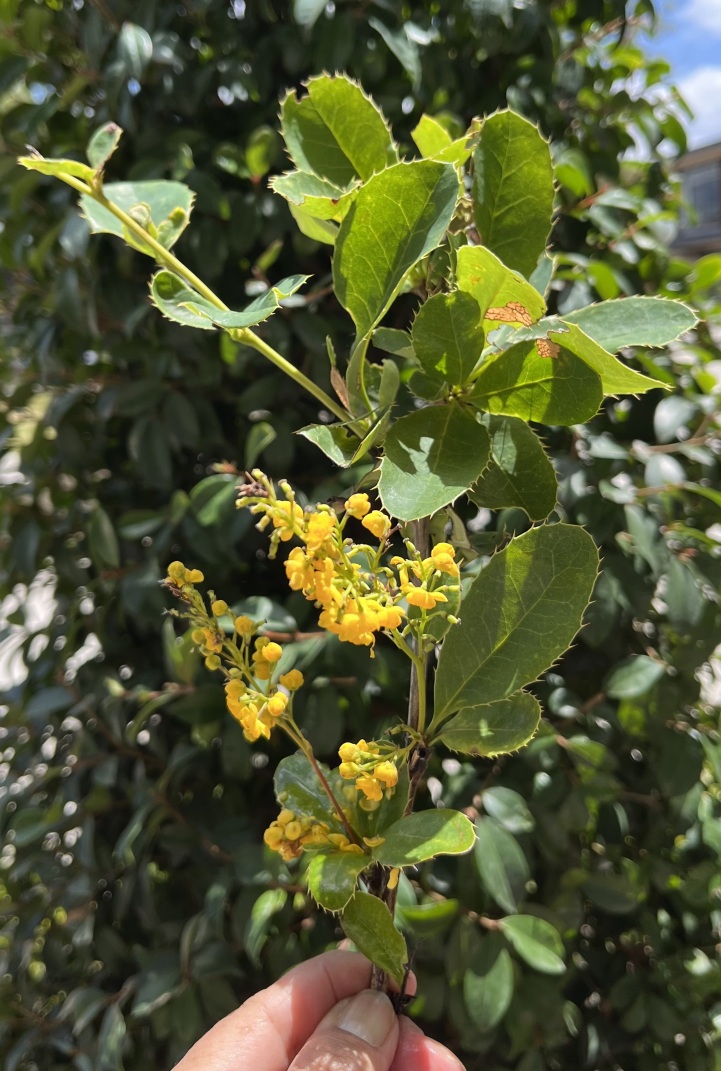
*Berberispectinata* at Azuay, Ecuador. Ulloa, C 2024. Photo courtesy Carmen Ulloa.

Figure(s) or Photo(s): Fig. 4

Region for assessment: Global

#### Editor & Reviewers

##### Editor

Editor: Ulloa Ulloa, C; Sánchez, E. & Oleas, N.H.

#### Geographic range

Biogeographic realm: Neotropical

Countries: Ecuador

Map of records (image): Fig. 1

Map of records (Google Earth): none

Basis of EOO and AOO: Observed

Basis (narrative): Unknown

Min Elevation/Depth (m): 1500

Max Elevation/Depth (m): 3000

Range description: Endemic species of Ecuador, recorded in at least five populations in the provinces of Chimborazo, Azuay and Loja. Additionally, its presence is confirmed in Parque Nacional Cajas.

#### Extent of occurrence

EOO (km2): 37

Trend: Unknown

Causes ceased?: Unknown

Causes understood?: Unknown

Causes reversible?: Unknown

Extreme fluctuations?: Unknown

#### Area of occupancy

Trend: Unknown

Causes ceased?: Unknown

Causes understood?: Unknown

Causes reversible?: Unknown

Extreme fluctuations?: Yes

Justification for extreme fluctuations: 

AOO (km2): 12

#### Locations

Number of locations: 3

Justification for number of locations: The species is documented in 3 locations distributed among the provinces of Azuay, Chimborazo and Loja.

Trend: Unknown

Extreme fluctuations?: Unknown

#### Population

Number of individuals: Unknown

Trend: Decline (observed)

Justification for trend: Continuing decline of mature individuals.

Causes ceased?: Unknown

Causes understood?: Unknown

Causes reversible?: Unknown

Extreme fluctuations?: Unknown

#### Subpopulations

Number of subpopulations: 3

Trend: Decline (observed)

Justification for trend: A subpopulation was previously known from Cimborazo in Ecuador, according to the assessment of this species in (Ulloa Ulloa and Pitman 2003b), but its record has been expanded.

Extreme fluctuations?: Unknown

Severe fragmentation?: Unknown

#### Habitat

System: Terrestrial

Habitat specialist: Yes

Habitat (narrative): High Andean forest, dry scrub, grasslands and livestock trails.

Trend in extent, area or quality?: Decline (observed)

Justification for trend: Continuing decline in area, extent and/or quality of habitat.

##### Habitat

Habitat importance: Major Importance

Habitats: 1. Forest1.9. Forest - Subtropical/Tropical Moist Montane4. Grassland4.7. Grassland - Subtropical/High Altitude

#### Ecology

Size: Unknown

Generation length (yr): 0

Dependency of single sp?: Unknown

Ecology and traits (narrative): Terrestrial

#### Threats

Justification for threats: The species is showing a continuing decline in area, extent, and/or habitat quality, and therefore a reduction in the number of mature individuals. This is probably due to road construction.

##### Threats

Threat type: Ongoing

Threats: 12. Other options - Other threat

#### Conservation

Justification for conservation actions:

Because both *B.pectinata* and *B.warszewiczii* were synonymized, the distribution of the species is very close to a protected area (Parque Nacional El Cajas). This finding highlights the potential presence of rare species in protected places that are important for the conservation of biodiversity.

**IUCN Red List assessment**

The number of mature individuals is declining, and the species has a limited distribution: extremely low EOO (37 km²) and very low AOO (12 km²), placing it in imminent danger of extinction. Furthermore, its habitat quality is continuously declining. Therefore, Berberispectinata is currently classified as '**Endangered' (EN)**, under criteria **B1ab (iii,v) + B2ab (iii,v).** The category could quickly be elevated to 'Critically Endangered' (CR) if urgent conservation measures are not taken.

##### Conservation actions

Conservation action type: Needed

Conservation actions: 1. Land/water protection1.2. Land/water protection - Resource & habitat protection2. Land/water management2.1. Land/water management - Site/area management3. Species management3.1. Species management - Species management5. Law & policy5.1. Law & policy - Legislation

#### Other

##### Use and trade

Use type: International

##### Ecosystem services

Ecosystem service type: Very important

##### Research needed

Research needed: 1. Research1.2. Research - Population size, distribution & trends1.3. Research - Life history & ecology1.5. Research - Threats2. Conservation Planning2.1. Conservation Planning - Species Action/Recovery Plan3. Monitoring3.1. Monitoring - Population trends3.3. Monitoring - Trade trends

Justification for research needed: It is necessary to develop research projects on the population dynamics and ecology of the species and in this way, provide the greatest amount of information possible for a better evaluation supported by several criteria.

### Berberis rigida

#### Species information

Scientific name: Berberisrigida

Species authority: Hieron.

Synonyms: *Berberislechleriana*

Kingdom: Plantae

Phylum: Streptophyta

Class: Equisetopsida

Order: Ranunculales

Family: Berberidaceae

Taxonomic notes: *Berberisrigida* includes *B.lechleriana* as a synonym, a poorly known name (Ulloa Ulloa and Oleas 2024). *Berberisrigida* is easily recognized by the obtrullate leaf blades with a cream-yellow venation above, forming a coarse reticulum and thickened margins. It is restricted to southern Ecuador.

**Figure 5.**
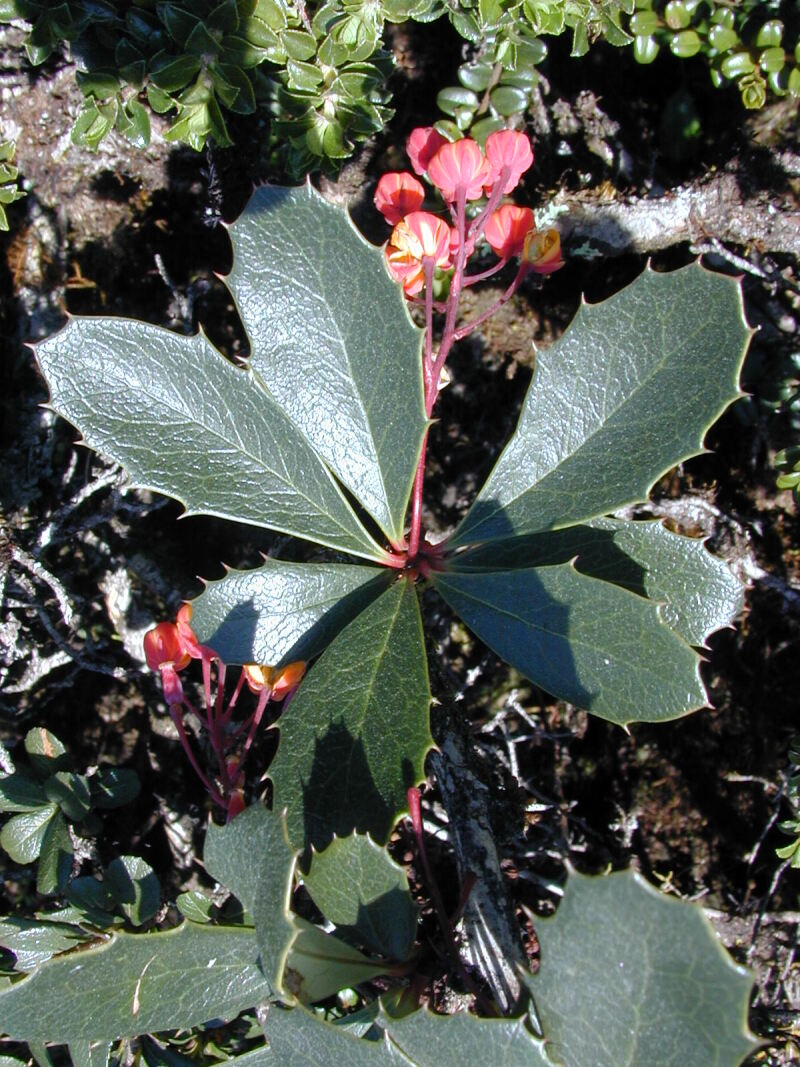
*Berberisrigida* at Ecuador. Ulloa, C. Photo courtesy Carmen Ulloa.

Figure(s) or Photo(s): Fig. 5

Region for assessment: Global

#### Editor & Reviewers

##### Editor

Editor: Ulloa Ulloa, C; Sánchez, E. & Oleas, N.H.

#### Geographic range

Biogeographic realm: Neotropical

Countries: Ecuador

Map of records (image): Fig. 1

Map of records (Google Earth): none

Basis of EOO and AOO: Observed

Basis (narrative): Unknow

Min Elevation/Depth (m): 2600

Max Elevation/Depth (m): 3950

Range description: Endemic species of Ecuador. The type locality was registered in Cuenca, Azuay. The collections come from the provinces of Azuay, Loja and Morona Santiago. Its presence is recorded in Parque Nacional Cajas and Parque Nacional Podocarpus.

#### Extent of occurrence

EOO (km2): 8409

Trend: Unknown

Causes ceased?: Unknown

Causes understood?: Unknown

Causes reversible?: Unknown

Extreme fluctuations?: Unknown

#### Area of occupancy

Trend: Unknown

Causes ceased?: Unknown

Causes understood?: Unknown

Causes reversible?: Unknown

Extreme fluctuations?: Unknown

AOO (km2): 48

#### Locations

Number of locations: 9

Justification for number of locations: The species is documented in nine locations distributed among the provinces of Azuay, Loja and Morona Santiago.

Trend: Increase

Justification for trend: A collection was known from a single locality in the province of Azuay, but it has now increased to 9.

Extreme fluctuations?: Unknown

#### Population

Trend: Unknown

Causes ceased?: Unknown

Causes understood?: Unknown

Causes reversible?: Unknown

Extreme fluctuations?: Unknown

#### Subpopulations

Number of subpopulations: 12

Trend: Unknown

Extreme fluctuations?: Unknown

Severe fragmentation?: Unknown

#### Habitat

System: Terrestrial

Habitat specialist: Yes

Habitat (narrative): It lives in *Polylepis* forests and páramos.

Trend in extent, area or quality?: Unknown

##### Habitat

Habitat importance: Major Importance

Habitats: 1. Forest1.9. Forest - Subtropical/Tropical Moist Montane

#### Ecology

Size: Unknown

Generation length (yr): 0

Dependency of single sp?: Unknown

Ecology and traits (narrative): Terrestrial

#### Threats

Justification for threats: Threat events for this species are unknown.

##### Threats

Threat type: Ongoing

Threats: 12. Other options - Other threat

#### Conservation

Justification for conservation actions:

This species has been collected in protected areas such as Parque Nacional Cajas and Parque Nacional Podocarpus. These findings highlight the importance of preserving these ecosystems, which serve as potential habitats for rare and endemic species.

**IUCN Red List assessment**

It could be classified as Vulnerable (VU) or even Endangered due to its extent of occurrence (EOO) and area of occupancy (AOO), which are well below the threshold. However, since no plausible threats are evident, such as a continued decline due to habitat fragmentation or possible future threats, it could be considered '**Near Threatened' (NT).**

##### Conservation actions

Conservation action type: Needed

Conservation actions: 1. Land/water protection1.1. Land/water protection - Site/area protection1.2. Land/water protection - Resource & habitat protection2. Land/water management2.1. Land/water management - Site/area management3. Species management3.1. Species management - Species management5. Law & policy5.1. Law & policy - Legislation5.2. Law & policy - Policies and regulations

#### Other

##### Use and trade

Use type: International

##### Ecosystem services

Ecosystem service type: Very important

##### Research needed

Research needed: 1. Research1.2. Research - Population size, distribution & trends1.5. Research - Threats2. Conservation Planning2.1. Conservation Planning - Species Action/Recovery Plan3. Monitoring3.1. Monitoring - Population trends3.3. Monitoring - Trade trends

Justification for research needed: It is necessary to develop research projects on the population dynamics and ecology of the species and in this way, provide the greatest amount of information possible for a better evaluation supported by several criteria.

### Berberis schwerinii

#### Species information

Scientific name: Berberisschwerinii

Species authority: C.K. Schneid.

Synonyms: *Berberischillacochensis*

Kingdom: Plantae

Phylum: Streptophyta

Class: Equisetopsida

Order: Ranunculales

Family: Berberidaceae

Taxonomic notes: *Berberisschwerinii* is unique among the Ecuadorian species of *Berberis* by the conspicuously glaucous stems, leaves, flowers and fruits. *Berberisschwerinii* includes *B.chillacochensis* as a synonym (Ulloa Ulloa and Oleas 2024).

Region for assessment: Global

#### Editor & Reviewers

##### Editor

Editor: Ulloa Ulloa, C; Sánchez, E. & Oleas, N.H.

#### Geographic range

Biogeographic realm: Neotropical

Countries: Ecuador

Map of records (image): Fig. 1

Map of records (Google Earth): none

Basis of EOO and AOO: Observed

Basis (narrative): Unknown

Min Elevation/Depth (m): 3000

Max Elevation/Depth (m): 3500

Range description: Endemic species of Ecuador. Few collections of this species are known and it has not been registered within the Sistema Nacional de Áreas Protegidas.

#### Extent of occurrence

EOO (km2): 33

Trend: Unknown

Causes ceased?: Unknown

Causes understood?: Unknown

Causes reversible?: Unknown

Extreme fluctuations?: Unknown

#### Area of occupancy

Trend: Unknown

Causes ceased?: Unknown

Causes understood?: Unknown

Causes reversible?: Unknown

Extreme fluctuations?: Unknown

AOO (km2): 12

#### Locations

Number of locations: 3

Justification for number of locations: *Berberisschwerinii* is a species endemic to Ecuador. There is a record of approximately five collections in the provinces of Azuay, El Oro, and Loja corresponding to three localities.

Trend: Unknown

Extreme fluctuations?: Unknown

#### Population

Trend: Unknown

Causes ceased?: Unknown

Causes understood?: Unknown

Causes reversible?: Unknown

Extreme fluctuations?: Unknown

#### Subpopulations

Number of subpopulations: 3

Trend: Unknown

Extreme fluctuations?: Unknown

Severe fragmentation?: Unknown

#### Habitat

System: Terrestrial

Habitat specialist: Yes

Habitat (narrative): It lives in humid grassland.

Trend in extent, area or quality?: Decline (observed)

Justification for trend: A continuous decline in habitat quality has been observed, primarily associated with the expansion of the agricultural frontier, arson, and degradation of the high Andean ecosystem.

##### Habitat

Habitat importance: Major Importance

Habitats: 4. Grassland4.7. Grassland - Subtropical/High Altitude

#### Ecology

Size: Unknown

Generation length (yr): 0

Dependency of single sp?: Unknown

Ecology and traits (narrative): Terrestrial

#### Threats

Justification for threats: Continuing decline in area, extent and/or quality of habitat, primarily associated with the expansion of the agricultural frontier, arson, and degradation of the high Andean ecosystem.

##### Threats

Threat type: Ongoing

Threats: 2. Agriculture & aquaculture11. Climate change & severe weather12. Other options - Other threat

#### Conservation

Justification for conservation actions:

There are no records of this species in Sistema Nacional de Areas Protegidas (Ulloa Ulloa and Pitman 2003a).

**IUCN Red List assessment**

Its extent of occurrence (EOO) is approximately 33 km² and its area of occupancy (AOO) is 12 km², both well below the thresholds established for the '**Endangered' (EN)** according to criterion **B1ab (iii)+2ab (iii)**. The species is known from only three locations, making it susceptible to stochastic events and fragmentation.

Furthermore, a continuous decline in habitat quality has been observed, primarily associated with the expansion of the agricultural frontier, arson, and degradation of the high Andean ecosystem.

##### Conservation actions

Conservation action type: Needed

Conservation actions: 1. Land/water protection1.1. Land/water protection - Site/area protection2. Land/water management2.1. Land/water management - Site/area management3. Species management3.1. Species management - Species management5. Law & policy5.1. Law & policy - Legislation

#### Other

##### Use and trade

Use type: International

##### Ecosystem services

Ecosystem service type: Very important

##### Research needed

Research needed: 1. Research1.2. Research - Population size, distribution & trends1.5. Research - Threats2. Conservation Planning2.1. Conservation Planning - Species Action/Recovery Plan3. Monitoring3.1. Monitoring - Population trends3.3. Monitoring - Trade trends

Justification for research needed: It is necessary to carry out sampling and searches for this species in other provinces of the country, as well as studies on population dynamics and threats. Provide as much information as possible for a better evaluation supported by several criteria.

## Discussion

The reassessment of conservation statuses for the *Berberis* species endemic to Ecuador reveals significant changes in threat levels for certain taxa, while others remain unchanged. The assessments consider factors such as the number of subpopulations, area of occupancy (AOO), extent of occurrence (EOO), and overall population trends.

Among the species that were previously not evaluated (Ulloa Ulloa and Pitman 2003a), *Berberisengleriana* is now classified as Endangered (EN), while *Berberisrigida* is assessed as Near Threatened (NT). These new evaluations highlight the importance of establishing baseline data for species previously lacking formal conservation status, allowing for targeted conservation efforts.

*Berberisjamesonii* is reclassified as Near Threatened (NT), indicating that habitat destruction is a plausible threat that could become a significant problem in the future. *Berberishyperythra* remains in the Data Deficient (DD) category, as no baseline information or images are available, making the available description insufficient to accurately assess its threat status.

Four species have changed their conservation status. The most critical change is observed in *Berberispectinata*, which was reclassified from DD to EN. Due to its small population and extremely limited distribution, the species is close to being classified as Critically Endangered (CR) if urgent conservation measures are not taken. Therefore, urgent population monitoring is suggested to prevent the taxon from reaching a higher threat category. *Berberisschwerinii* was reclassified from DD to EN, suggesting that available information and studies on these species threats and distribution significantly increase the risk level.

*Berberislaidivo* remains in the DD category due to very high uncertainty, as no plausible future serious threat has been documented that would cause the species to rapidly decline to EN or CR. *Berberisminzaensis* has been reassessed from Near Threatened (NT) to Vulnerable (VU) due to a possible population decline caused by ongoing habitat destruction. These factors, together, indicate a possible increased risk of extinction, warranting urgent conservation action.

According to the present assessment, three of the seven *Berberis* species endemic to Ecuador occur within Protected Areas. This underscores the urgent need to expand these conservation areas to safeguard endangered species facing widespread habitat destruction.

The creation of new protected areas is a fundamental step to mitigate habitat loss and fragmentation, as well as to safeguard ecosystems, species, and even the preservation of traditional cultures (Mestanza-Ramón et al. 2023). In Ecuador, this strategy is particularly relevant due to the accelerated development of infrastructure within and around protected areas, which could exert considerable pressure and contribute to habitat fragmentation (González-Salas et al. 2024). The main conservation instrument in the country is the National System of Protected Areas (SNAP), which currently recognizes 74 areas under different forms of management: state, decentralized autonomous, community, and private (Cuesta et al. 2013;González-Salas et al. 2024). As of 2021, fourteen new protected areas have been incorporated into the SNAP, thus expanding the scope of this national conservation system (Mestanza-Ramón et al. 2020). Despite this, many populations remain under constant threat due to the ongoing degradation of páramos, making them increasingly vulnerable, as reflected in this study.

Our research also highlights the crucial role of herbarium collections and taxonomic revisions in advancing the study of Andean flora, especially those collections made many years ago, which lack sufficient updated information and have not been compiled recently. An example of this study is the situation of *Berberislaidivo* and the need to monitor this species to address the information gap. However, there remains a significant gap in fundamental ecological knowledge of these species, including aspects such as generation length, population size, and trends in population dynamics. To effectively conserve and sustainably manage these ecosystems, it is essential to understand patterns of genetic diversity among the unique species of the Andes. The lack of studies on the demography and population structure of *Berberis* species underlines the urgent need for comprehensive inventories. Additionally, disseminating existing data and relevant information is vital to facilitate updated reassessments of these species' conservation statuses.

Discussion

## Figures and Tables

**Table 1.** This table shows those species of *Berberis* that have been evaluated because they were considered endemic to Ecuador, following IUCN (International Union for Conservation of Nature) criteria. **LC**: Least Concern, **VU**: Vulnerable, **EN**: Endangered, **NT**: Near Threatened, **DD**: Data Deficient. The **Change** column indicates whether the species status has been updated based on new data and changes in taxonomy.

Species	Initial Assessment Ulloa Ulloa and Pitman 2003e, Ulloa Ulloa and Pitman 2003d, Ulloa Ulloa and Pitman 2003c, Ulloa Ulloa and Pitman 2003b, Ulloa Ulloa and Pitman 2003a	Accepted name in Ulloa Ulloa and Oleas (2024)	Proposed Assessment	Change based on information in Ulloa Ulloa and Oleas (2024)
-	-	*Berberisengleriana*	EN	New evaluation. Evaluated in the EN category.
*Berberischillacochensis*	DD	*Berberisschwerinii*	EN	Changed to EN category. Taxonomic change: *B.chillacochensis* is a synonym of *B.schwerinii*.
*Berberisfarinosa*	DD	*Berberispichinchensis*	-	Taxonomy change: Synonym of *B.pichinchensis* (not endemic, also present in South of Colombia).
*Berberishirtellipes*	DD	*Berberispichinchensis*	-	Taxonomy change: Synonym of *B.pichinchensis* (not endemic, also present in South of Colombia).
*Berberishyperythra*	DD	*Berberishyperythra*	DD	No change in the category. Incompletely known species.
*Berberisjamesonii*	LC	*Berberisjamesonii*	NT	Changed to NT category, due to its number of locations.
*Berberislaidivo*	DD	*Berberislaidivo*	DD	Present in only 2 locations without further information.
*Berberislechleriana*	DD	*Berberisrigida*	NT	Changed to NT category. Due to a taxonomic change, currently there are 12 subpopulations. Taxonomy change: *B.lechleriana* is a synonym of *B.rigida*.
*Berberisminzaensis*	NT	*Berberisminzaensis*	VU	Changed to VU category. A possible significant population decline due to habitat destruction and ongoing threats. More information is needed.
*Berberispapillosa*	DD	*Berberisgrandiflora*	-	Taxonomy changes: Synonym of *B.grandiflora* (not endemic, present from Colombia to Peru).
*Berberispavoniana*	DD	*Berberislutea*	-	Taxonomy changes: Synonym of *B.lutea* (not endemic, present from Colombia to Bolivia).
*Berberispectinata*	DD	*Berberispectinata*	EN	Changed to EN category. Small number of locations and extremely limited distribution. The species is close to being classified as Critically Endangered (CR) if urgent conservation measures are not taken. Need monitoring. Taxonomy changes: New synonym is *B.warszewiczii*; this name was not evaluated before
*Berberispindilicensis*	VU	*Berberismultiflora*	-	Taxonomy changes: Synonym of *B.multiflora* (not endemic, also present in North of Peru).
*Berberisreicheana*	DD	*Berberispichinchensis*	-	Taxonomy changes: Synonym of *B.pichinchensis* (not endemic, also present in South of Colombia).
*Berberissaxorum*	DD	*Berberispichinchensis*	-	Taxonomy changes: Synonym of *B.pichinchensis* (not endemic, also present in South of Colombia).
*Berberissimonsii*	DD	*Berberispichinchensis*	-	Taxonomy changes: Synonym of *B.pichinchensis* (not endemic, also present in South of Colombia).
